# Enhanced MRI-PET fusion using Laplacian pyramid and empirical mode decomposition for improved oncology imaging

**DOI:** 10.1371/journal.pone.0322443

**Published:** 2025-05-19

**Authors:** Gunnam Suryanarayana, Satyanarayana Murthy Nimmagadda, Sabbavarapu Nageswara Rao, Ali Mohammed Y. Mahnashi, Shri Ramtej Kondamuri, Abdullah Ali Hussain Ahmadini, Abdullah Ahmed Zaagan, Ahmed Hussein Msmali

**Affiliations:** 1 Department of Electronics and Communication Engineering, Siddhartha Academy of Higher Education, Deemed to be University, Vijayawada, Andhra Pradesh, India; 2 Department of Mathematics, College of Science, Jazan University, Jazan, Kingdom of Saudi Arabia; University of Electronic Science and Technology of China, CHINA

## Abstract

In the field of oncology imaging, the fusion of magnetic resonance imaging (MRI) and positron emission tomography (PET) modalities is crucial for enhancing diagnostic capabilities. This article introduces a novel fusion method that leverages the strengths of both modalities to overcome limitations associated with functional information in MRI and the spatial resolution in PET scans. Our approach integrates the Laplacian pyramid for extracting high and low-frequency components, along with empirical mode decomposition and phase congruency to preserve crucial structural details in the fused image. Additionally, a rolling guidance filter is employed to mitigate edge detail loss. Through extensive comparative experiments on multi-focus and multi-modal image datasets, our method consistently outperforms existing techniques in terms of visualization, objective metrics, and computational efficiency. The proposed fusion method demonstrates superior performance, establishing it as a compelling alternative for oncology imaging applications.

## 1. Introduction

In oncology imaging, the combination of magnetic resonance imaging (MRI) and positron emission tomography (PET) plays a pivotal role in detecting conditions such as breast cancer and brain tumors. While MRI offers high-resolution anatomical details, it often lacks depth in functional information. Conversely, PET provides functional information but with lower spatial resolution, posing challenges for accurate disease diagnosis [1]. To address these limitations and harness the complementary strengths of both modalities, image fusion techniques have been widely explored. Image fusion involves combining information from multiple source images to create a single composite image that integrates the best features of each modality [2]. Various fusion methods can be categorized into spatial domain-based, transform domain-based, and learning-based techniques.

Spatial domain methods directly manipulate the individual pixels within the input image to achieve the desired result [3]. This method does not require sophisticated mathematical procedures or frequency domain conversions. The most popular techniques for spatial fusion include weighted average, principal component analysis, and simple fusion methods [4]. While these techniques are user-friendly, they often yield fused images with notable distortion and reduced effectiveness.

Transform domain fusion involves the conversion of input images from the spatial domain to the frequency domain using various transform methods such as hybrid transforms, Stationary Wavelet Transform (SWT), Discrete Cosine Transform (DCT), and Discrete Wavelet Transform (DWT) [4–8]. This process entails partitioning the input image into numerous sub-band images through low-pass and high-pass filtering [9]. Transform domain fusion methods typically result in improved peak signal-to-noise ratios (PSNR) and reduced mean square errors (MSE). However, they are often attributed to translational invariance, which can potentially lead to distorted edges in the output image [10].

Learning-based techniques utilize machine learning or deep learning algorithms to automatically learn the fusion process from a set of training data [11–14]. Convolutional Neural Networks (CNNs) are commonly used for learning-based fusion tasks [15,16]. These networks learn hierarchical representations of input images and fuse them at different levels of abstraction to generate the fused image. Learning-based fusion techniques have shown promising results in various applications and can adapt to different types of input data [17,18]. They are particularly useful when the fusion problem is complex and traditional techniques may not be sufficient.

However, learning-based techniques often require large datasets, which may not always be practical in medical scenarios. Additionally, these methods tend to be computationally intensive compared to spatial and transform domain techniques. Therefore, in this work, we propose a novel MRI-PET fusion method using a combination of Laplacian pyramid (LP) and empirical mode decomposition (EMD). This approach aims to extract and integrate high and low-frequency components effectively while preserving crucial structural details within the fused image. Our method achieves computational balance by applying phase congruency (PC) and rolling guidance filter (RGF) exclusively on the intrinsic mode functions of high-frequency components. Unlike existing methods, our approach strikes a balance between computational efficiency and information preservation, making it suitable for medical image fusion tasks.

Section 2 provides an overview of the preliminary techniques utilized in this study, including the RGF and LP. The proposed fusion method is detailed in Section 3, where we outline our approach for combining these techniques to achieve effective image fusion. In Section 4, we validate our proposed method through both qualitative and quantitative analyses, evaluating its performance against established benchmarks. Finally, Section 5 concludes the paper, summarizing key findings.

## 2. Preliminaries

This section provides an overview of the RGF and LP techniques used in this work.

### 2.1. Rolling guidance filter

RGF is a pivotal element within our proposed method for fusing MRI and PET images. Its primary function is to counteract the loss of edge details that often occurs during the fusion process [19]. Unlike conventional filtering techniques, the RGF dynamically adjusts to the local structures present in the images, thereby preserving crucial edge information. This adaptability contributes significantly to achieving higher peak signal-to-noise ratios (PSNR) and lower mean square errors (MSE) compared to previous fusion methodologies especially in transform domain. By retaining edge details, the RGF substantially improves the overall quality of the fused images, thereby addressing the challenges posed by the low spatial resolution of PET and the limited functional information provided by MRI [20].

**Fig 1:**
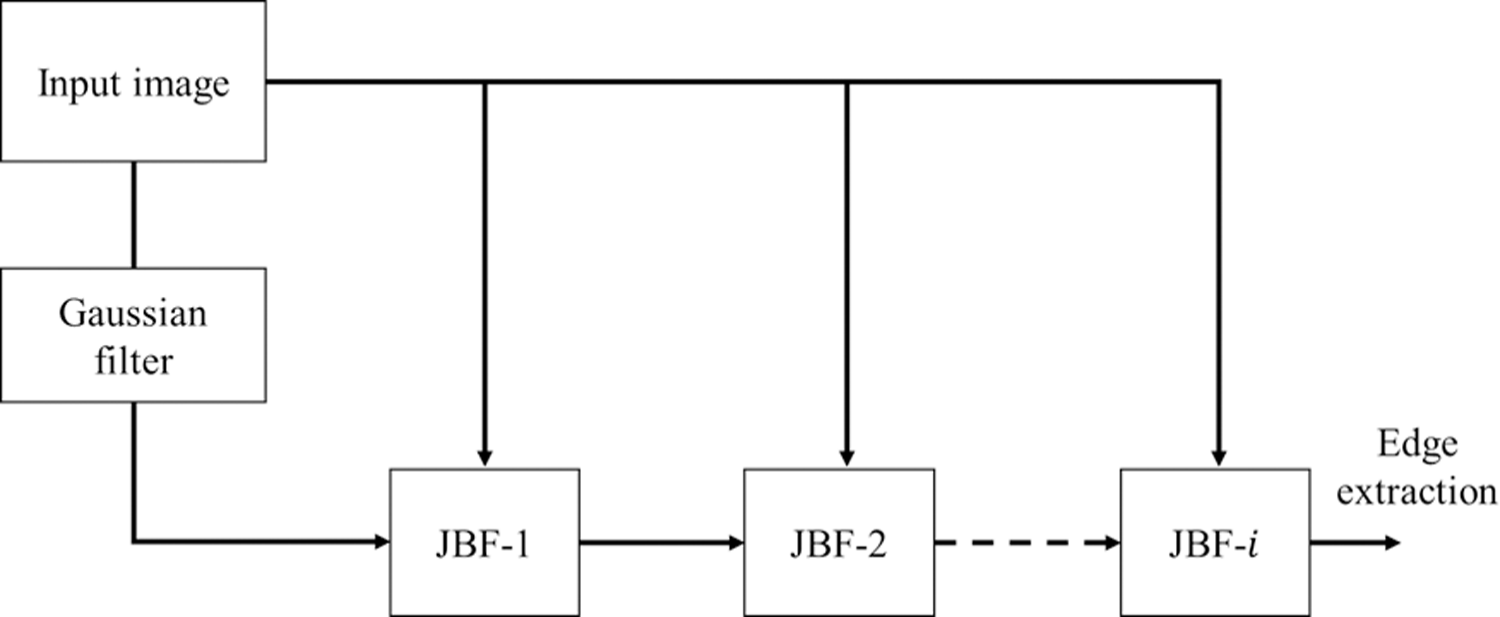
Recursive Gaussian and JBF operation in RGF.


RGF integrates essential components such as the Gaussian filter (GF) and the joint bilateral filter (JBF). The block diagram of RGF shown in Fig 1 illustrates the input passing through a GF followed by i stages of JBFs. The output of GF is denoted by G[p, q], while the output of ith stage JBF is denoted by $J^{i+1}\left[p,q\right]$. These filters [21] are implemented in the spatial domain using moving window operation as

$$\begin{array}{c} \begin{array}{c} Y\left[p,q\right]=\frac{\sum_{\left(r,s\right)\in Z\left(p,q\right)}X\left[r,s\right]W\left[p,q;r,s\right]}{\sum_{\left(r,s\right)\in Z\left(p,q\right)}W\left[p,q;r,s\right]}. \end{array} \end{array}$$ (1)

In the equation, (r, s) denotes the index pixel coordinates, while Z(p,q) represents the neighborhood surrounding the (p,q)th pixel. X[r,s] is the pixel intensity at (r, s) in the input image, serving as the initial data to be processed by the RGF. Y[p,q] represents the filtered output pixel value at (p,q). The weight assigned to the (r, s)th input pixel in the (p,q)th output pixel is denoted by W[p,q;r,s]. For Gaussian filter, the weight is determined by


$$\begin{array}{c} W\left[p,q;r,s\right]=exp\left\{-\frac{{\left(p-r\right)}^{2}+{\left(q-s\right)}^{2}}{2\sigma_{s}^{2}}\right\}. \end{array}$$ (2)

From scale space theory, the GF smoothens details, with the scale parameter dependent on the degree of high-contrast edges intended to be preserved. However, the GF not only removes small-scaled structures but also blurs large-scaled structures. To recover these blurred high-contrast edges, the Gaussian output is iteratively filtered using JBF, with weights determined by the equation


$$\begin{array}{c} \begin{array}{c} W\left[p,q;r,s\right]=exp\left\{-\frac{{\left(p-r\right)}^{2}+{\left(q-s\right)}^{2}}{2\sigma_{s}^{2}}-\frac{\|J^{i}\left[p,q\right]-J^{i}{\left[r,s\right]\|}^{2}}{2\sigma_{r}^{2}}\right\}. \end{array} \end{array}$$ (3)

Here, the first iteration starts with $J^{1}=G$. JBF considers both Euclidean distance and radiometric differences, recovering high-contrast edges by adjusting weights according to neighbouring pixels. Additionally, the degree of sharpness increases with each stage of filtering due to the cascading effect of these filters.

Indeed, $\sigma_{s}$ and $\sigma_{r}$ play crucial roles in controlling the spatial and range weights, respectively, within the JBF. These parameters determine the extent of spatial and intensity variations considered during the filtering process. By utilizing the input image and previous Gaussian functions, the JBF effectively generates images that retain Gaussian edge information while simultaneously preserving structural details and minimizing artifacts. This approach ensures that the filtering process maintains edge sharpness and overall image quality.

### 2.2. Laplacian pyramid

The LP, pioneered by Burt et al [22], marks a significant milestone in image processing and computer vision. This hierarchical image representation method is renowned for its efficiency and adaptability across a range of image analysis tasks. Unlike conventional pyramidal structures, the LP doesn’t store down-sampled versions of the image. Instead, it innovatively decomposes the original image into a sequence of successively down-sampled and band-pass-filtered sub-images, each capturing distinct frequency components. This approach enables the extraction of intricate details while preserving computational resources. One of its key strengths lies in providing a multiresolution representation of the image. Each level of the pyramid corresponds to a specific frequency band, facilitating the independent analysis of various features [23]. This hierarchical structure proves invaluable in scenarios demanding detailed examination of specific frequency components.

**Fig 2:**
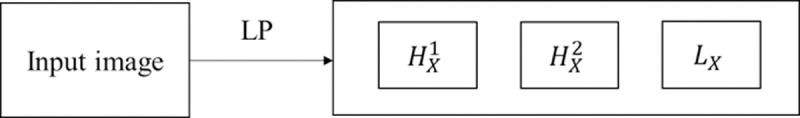
Laplacian Pyramid decomposition.

The decomposition of input image X is depicted in Fig 2, where $H_{X}^{1}$ and $H_{X}^{2}$ represent high frequency sub-band images, while $L_{X}$ denotes the low frequency sub-band image. The LP is widely employed in image compression, excelling at capturing and preserving visual details while reducing overall data size, making it invaluable for storage or bandwidth-constrained applications. Additionally, it is highly effective in edge detection tasks, emphasizing fine features crucial for feature identification in computer vision applications. Moreover, the LP is pivotal in image blending, seamlessly integrating different images at various levels to produce a blended output that retains essential details from each source image. This capability is particularly useful in graphics and visual effects, where smooth transitions are paramount. Supporting progressive image reconstruction, the LP enables obtaining approximations of the original image at different resolutions, catering to scenarios requiring both quick overviews and detailed refinement [24]. Overall, it stands as a cornerstone in image processing and computer vision, offering a compact, multiresolution representation that continues to shape advancements in the field.

By integrating RGF and LP, the proposed fusion approach not only leverages the strengths of multi-scale decomposition and selective smoothing but also ensures that both high and low-frequency components are optimally processed.

## 3. Proposed method

**Fig 3:**
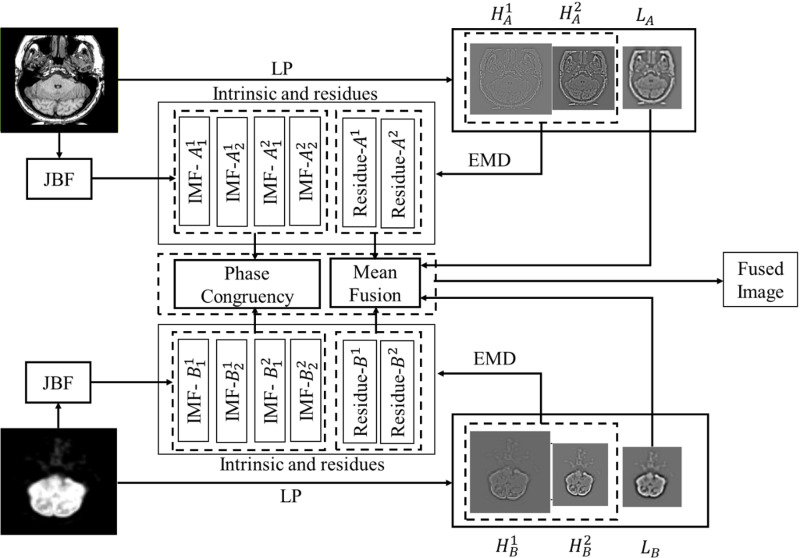
Schematic view of the proposed MRI-PET fusion.

In this section, we delve into the detailed processes of PC, EMD, and the LP, considering an input MRI image A and a PET image B. The block diagram of proposed method is depicted in Fig 3.

*Laplacian Pyramid-* Application of LP to the input images generates sub-band images at both high and low frequencies. For image A, this results in


$$\begin{array}{c} LP\left(A\right)\to \left[H_{A}^{1}+H_{A}^{2}+L_{A}\right], \end{array}$$ (4)

where $H_{A}^{1}$, $H_{A}^{2}$ represent sub-band images at high frequencies, and $L_{A}$ denotes the low-frequency sub-band image. Similarly, for image B


$$\begin{array}{c} LP\left(B\right)\to \left[H_{B}^{1}+H_{B}^{2}+L_{B}\right], \end{array}$$ (5)

where $H_{B}^{1},H_{B}^{2}$ represent sub-band images at high frequencies, and $L_{B}$ denotes the low-frequency sub-band image.

*Empirical mode decomposition-* Applying EMD to the high-frequency sub-band images generates residue as well as intrinsic mode functions (IMFs). In this context, the residue exhibits low frequency, while the IMFs demonstrate high frequency.


$$\begin{array}{c} EMD\left(H_{A}^{1}\right)\to \left[IMF-A_{1}^{1},\;\;\;\;\;\;\;IMF-A_{2}^{1},\;\;\;\;\;\;\;RES-A^{1}\right], \end{array}$$ (6)

$$\begin{array}{c} EMD\left(H_{A}^{2}\right)\to \left[IMF-A_{1}^{2},\;\;\;\;\;\;\;IMF-A_{2}^{2},\;\;\;\;\;\;\;RES-A^{2}\right], \end{array}$$ (7)

$$\begin{array}{c} EMD\left(H_{B}^{1}\right)\to \left[IMF-B_{1}^{1},\;\;\;\;\;\;\;IMF-B_{2}^{1},\;\;\;\;\;\;\;RES-B^{1}\right], \end{array}$$ (8)

$$\begin{array}{c} EMD\left(H_{B}^{2}\right)\to \left[IMF-B_{1}^{2},\;\;\;\;\;\;\;IMF-B_{2}^{2},\;\;\;\;\;\;\;RES-B^{2}\right]. \end{array}$$ (9)

Equation (6) represents the decomposition of $H_{A}^{1}\;\;\;\;\;\;\;$into IMFs $A_{1}^{1},A_{2}^{1}$ and residual function $A^{1}$ using EMD. In equation (7), $A_{1}^{2},A_{2}^{2}$, and $A^{2\;\;\;\;\;\;\;}$are the IMFs and residue of $H_{A}^{2}$. Similarly, $B_{1}^{1},B_{2}^{1}$, and $B^{1\;\;\;\;\;\;\;}$ corresponds to $H_{B}^{1}$, while $B_{1}^{2},B_{2}^{2}$, and $B^{2\;\;\;\;\;\;\;}$ corresponds to $H_{B}^{2}$. Following decomposition, all these IMFs undergo edge extraction using Gaussian and JBF operations.

*Phase congruency*- Leveraging the phase congruence of Fourier components, PC is capable of highlighting small discontinuities in images with varying lighting and contrast, thereby aiding in a deeper understanding of the image’s context and content. This approach detects subtle variations in image intensity and highlights anomalous values. This fusion process of IMFs based on PC can be described as


$$\begin{array}{c} PC\left(IMF-A_{1}^{1},IMF-B_{1}^{1}\right)\to IMF-F_{1}^{1}, \end{array}$$ (10)

$$\begin{array}{c} PC\left(IMF-A_{1}^{2},IMF-B_{1}^{2}\right)\to IMF-F_{1}^{2}, \end{array}$$ (11)

$$\begin{array}{c} PC\left(IMF-A_{2}^{1},IMF-B_{2}^{1}\right)\to IMF-F_{2}^{1}, \end{array}$$ (12)

$$\begin{array}{c} PC\left(IMF-A_{2}^{2},IMF-B_{2}^{2}\right)\to IMF-F_{2}^{2}. \end{array}$$ (13)

Here $F_{1}^{1},\;F_{1}^{2}$ are the fused IMFs corresponding to $\left(A_{1}^{1},B_{1}^{1}\right)$ and $(A_{1}^{2},B_{1}^{2}),$ respectively. $F_{2}^{1},\;F_{2}^{2}$ are the respective fused IMFs corresponding to $\left(A_{2}^{1},B_{2}^{1}\right)$ and $\left(A_{2}^{2},B_{2}^{2}\right)$.

*Mean fusion*- Mean fusion (MF) is applied to the residues $RES-A^{1}$ and $RES-B^{1}$ to yield $RES-F^{1}$, and similarly to $RES-A^{2}$ and $RES-B^{2}$ to produce $RES-F^{2}$. The low-frequency sub-band images $L_{A},L_{B}$ corresponding to MRI and PET images are also subjected to mean fusion resulting in $F^{3}.$


$$\begin{array}{c} MF\left(RES-A^{1},RES-B^{1}\right)\to RES-F^{1}, \end{array}$$ (14)

$$\begin{array}{c} MF\left(RES-A^{2},RES-B^{2}\right)\to RES-F^{2}, \end{array}$$ (15)

$$\begin{array}{c} MF\left(L_{A},L_{B}\right)\to F^{3}. \end{array}$$ (16)

Finally, the resultant fused image *F* is produced by combining the fused IMFs $F_{1}^{1},\;F_{1}^{2},\;F_{2}^{1},$ and $F_{2}^{2},$ along with the residuals $F^{1},\;F^{2},$ and the low-frequency sub-band $F^{3}.$


$$\begin{array}{c} IMF-F_{1}^{1}+IMF-F_{2}^{1}+RES-F^{1}+IMF-F_{1}^{2}+IMF-F_{2}^{2}+RES-F^{2}+F^{3}\to F. \end{array}$$ (17)

The fused image F exhibits enhanced structural detail preservation and edge clarity, offering a robust solution for advancing diagnostic precision and efficacy in oncology imaging.

## 4. Experiments and results

This section outlines the fusion process applied to diverse datasets, evaluating algorithmic performance using key metrics such as peak signal-to-noise ratio (PSNR), structural similarity index (SSIM), mutual information (MI), average gradient (AG), and entropy fusion (EN), quality of edge information (QABF), loss of edge information (LABF), and noise or artifacts added during the fusion (NABF). The sum of QABF, LABF, and NABF should be unity. A comparative analysis was conducted to assess the effectiveness of our algorithm against alternatives, including structure-aware (STAW) [12], nonsubsampled contourlet transform (NSCT) [5], Laplacian re-decomposition (LRD) [6], CNN [13], EMD [7], inter-modality attention (IMA) [14], dual-tree complex wavelet transform (DTCWT) [8], correlation-driven feature decomposition fusion, FCDFusion [25], CDDFuse [26], and CMTFusion [27]. All experiments and output visualizations were exclusively performed in MATLAB 2021 on a laptop equipped with an Intel Core i4 CPU and 4.0 GB RAM. We used two datasets to evaluate different fusion methods. Dataset-1 comprises 10 MRI-PET test image pairs [28], while dataset-2 contains 269 image pairs [29]. From dataset-2, 245 pairs were used to train the learning-based models, and the remaining 24 image pairs were used for evaluation.

**Fig 4:**
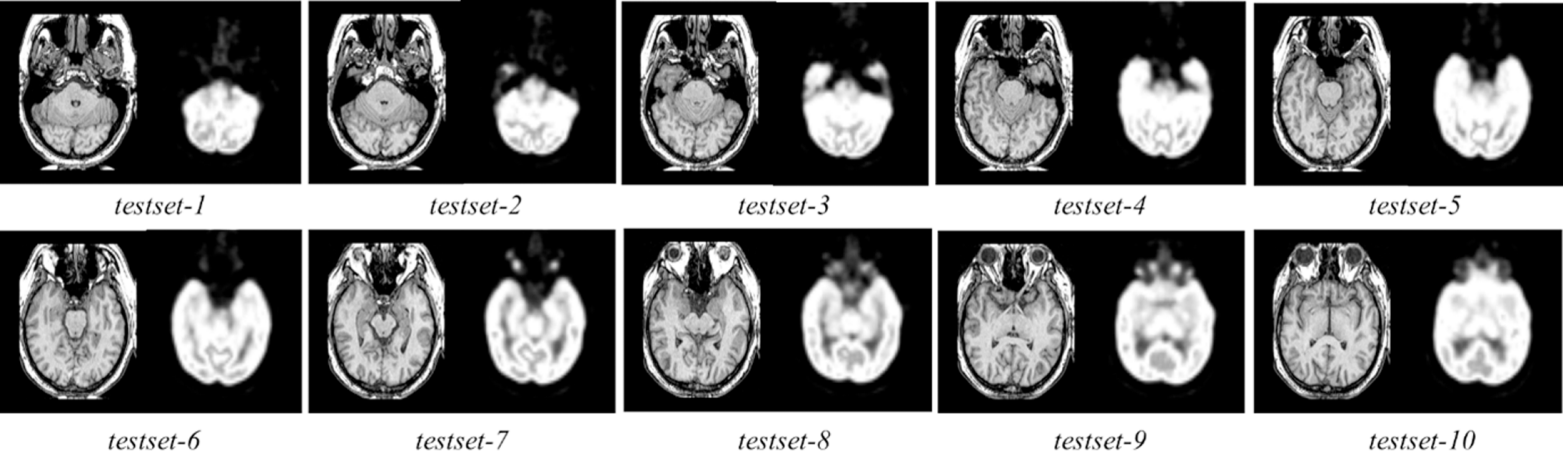
Evaluation dataset-1 for MRI-PET images.

**Fig 5:**
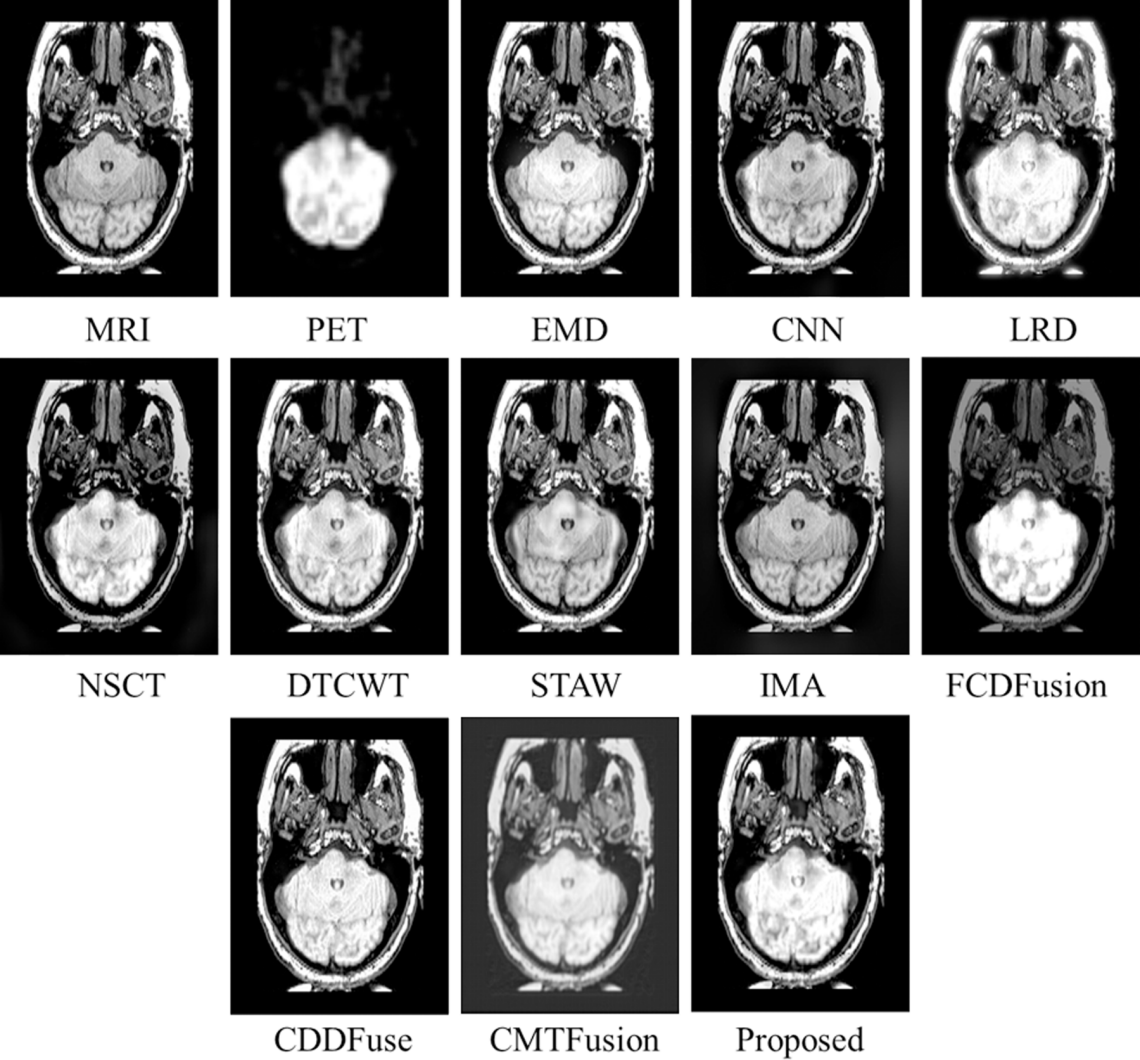
Fusion results of various techniques on *testset-1.*

Fig 4 presents the test MRI and PET images [28] used for evaluation of various fusion methods. The fusion results of the proposed technique and other existing methods on MRI-PET *testset-1* are displayed in Fig 5. The initial two images depict the MRI-PET source images, followed by the fusion results generated by EMD, CNN, LRD, NSCT, DTCWT, STAW, IMA, FCDFusion, CCDFuse, CMTFusion and our proposed technique. While most techniques excel in capturing structural details from the MRI, distinctions arise in edge extraction quality. Notably, EMD, CNN, LRD, NSCT, DTCWT, STAW, and IMA exhibit significant edge distortion in the fusion results, with varying degrees of local detail loss in the image. Although EMD and LRD successfully preserve edge information, they tend to overly enhance spatial structure in the MRI, leading to some original information loss. In contrast, our proposed fusion method excels in both fidelity and visual quality. It achieves higher definition in local details of the fused image while maintaining overall structural integrity.

**Fig 6:**
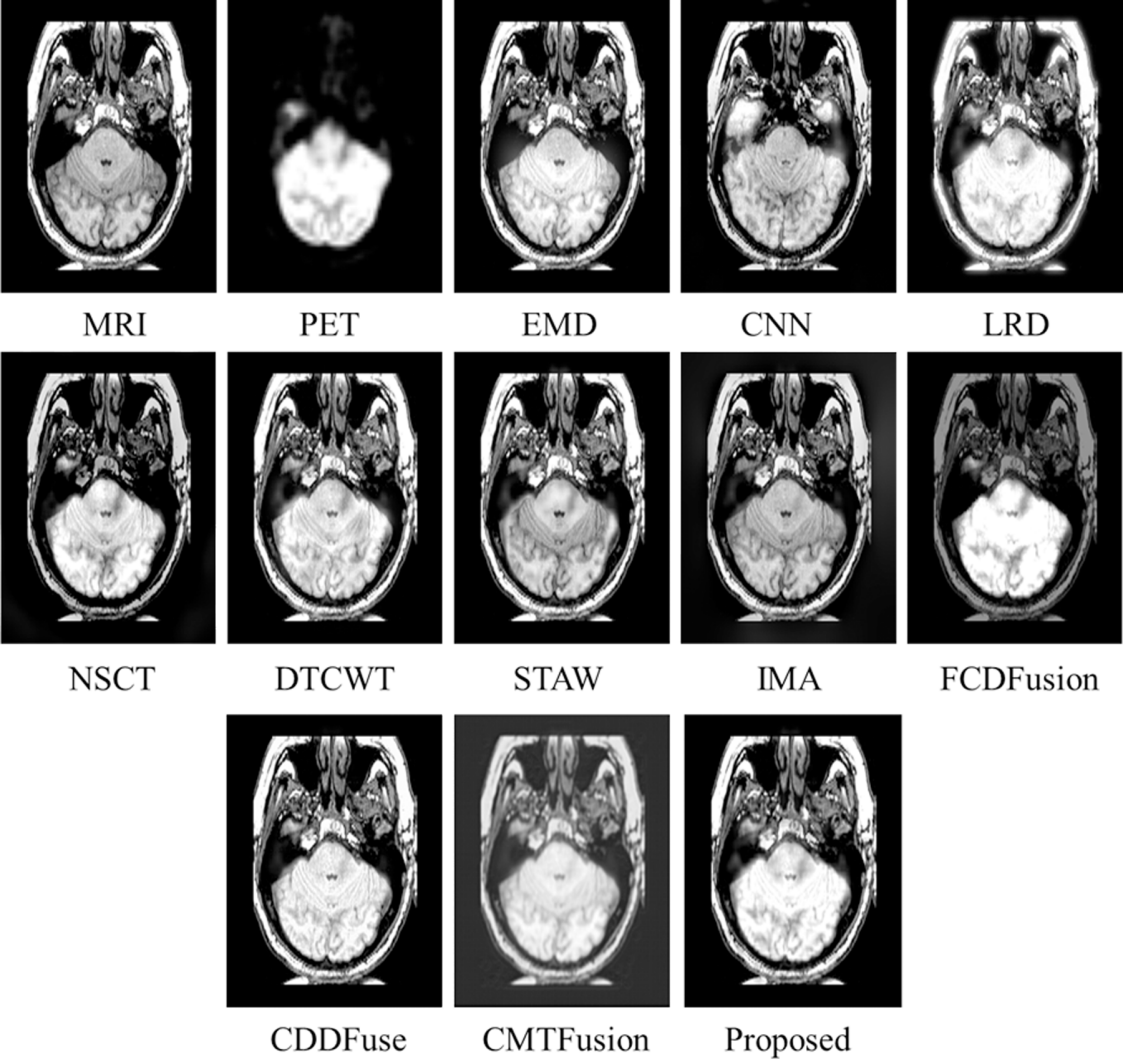
Fusion results of various techniques on *testset-2.*

**Fig 7:**
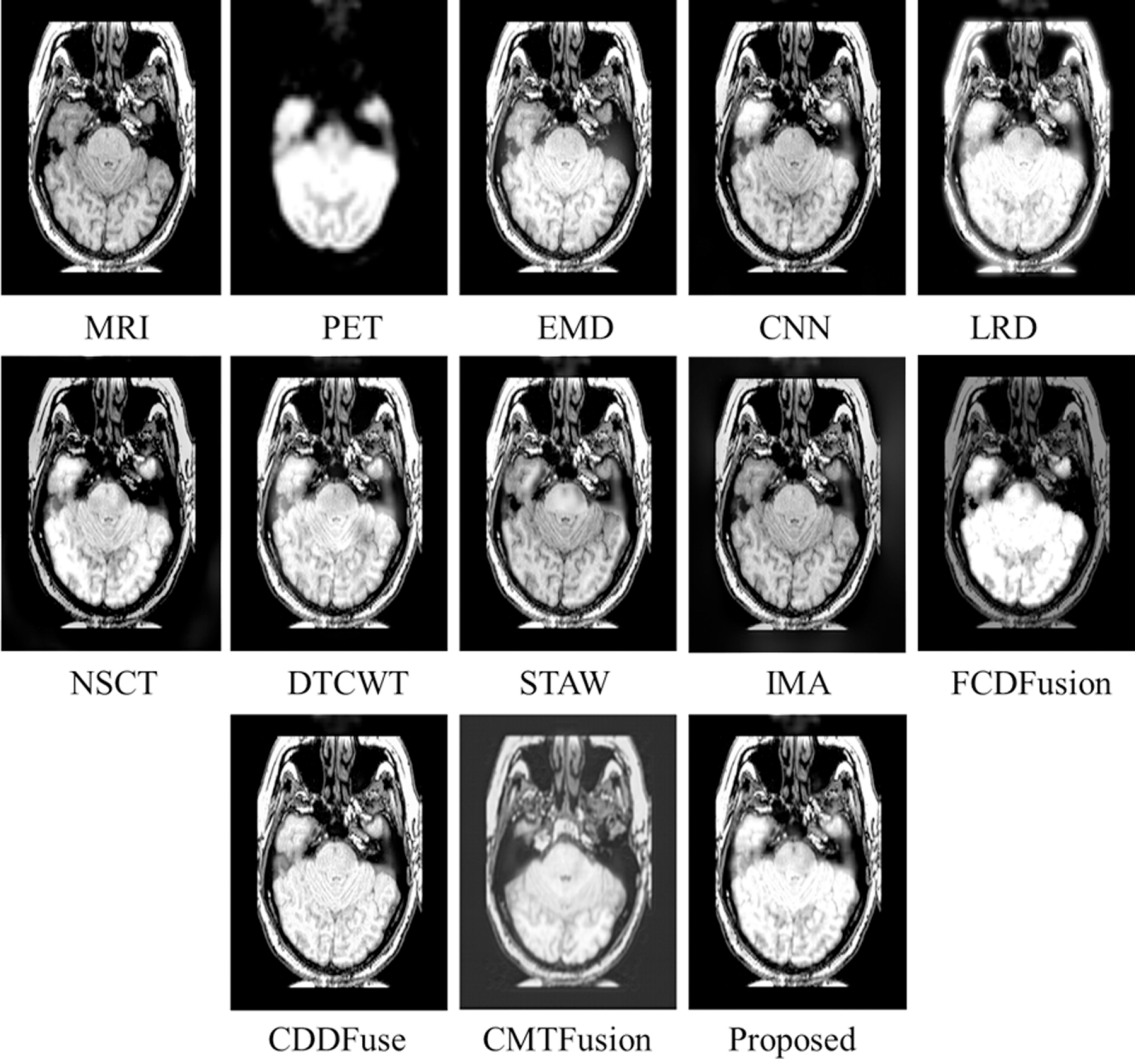
Fusion results of various techniques on *testset-3.*

**Fig 8:**
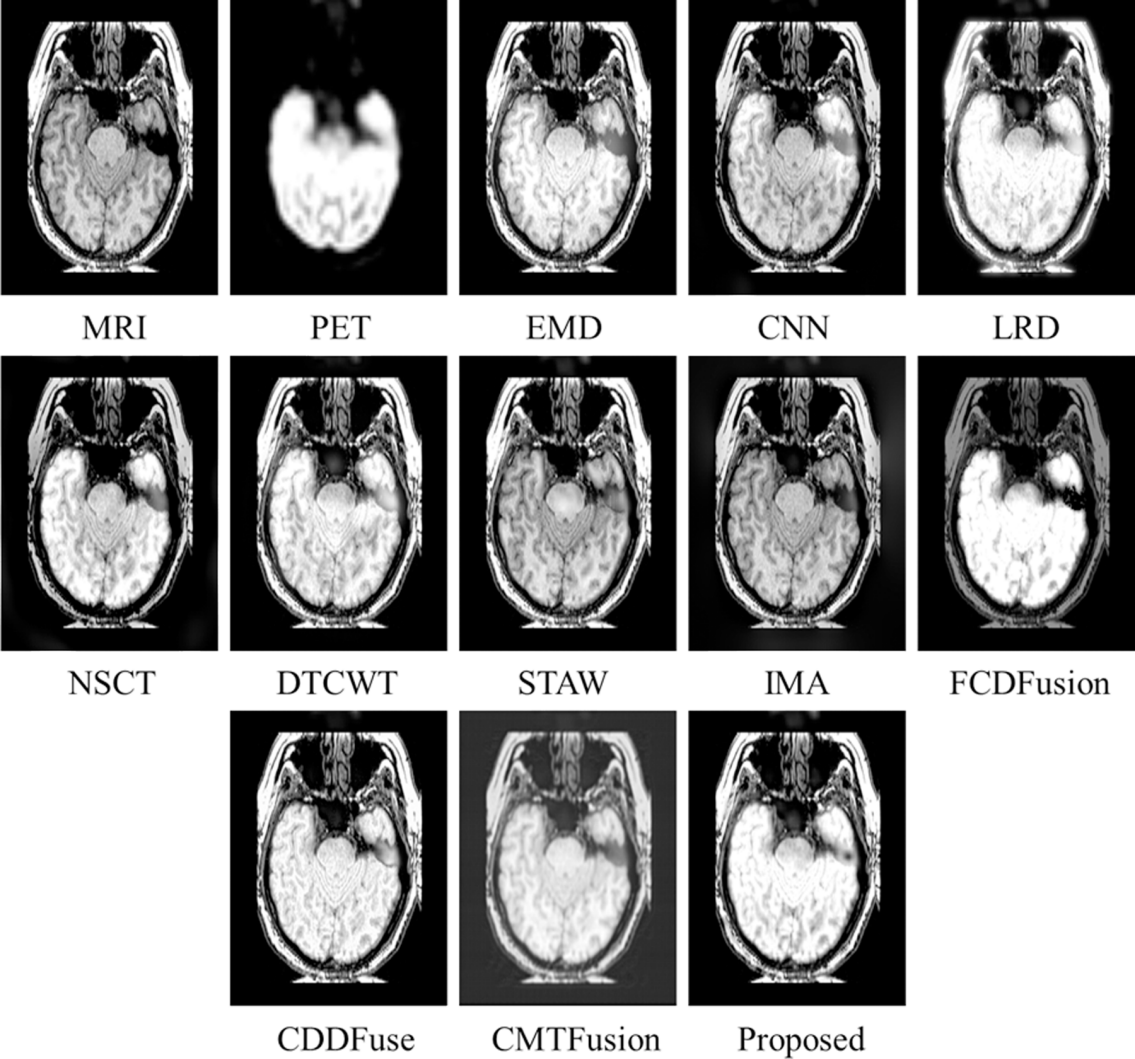
Fusion results of various techniques on *testset-4.*

Similarly, Figs 6–8 present fusion results of various techniques on MRI-PET *testset-2*, *testset-3*, and *testset-4* respectively, providing a comparative analysis of the effectiveness of various fusion methods in capturing and preserving important information from the input datasets.

Table 1 presents a comparison of various image fusion methods based on different evaluation metrics for image pairs taken from [28]. Ideally, EN, PSNR, MI, and AG should have high values, indicating better image quality, information retention, and sharpness. QABF and SSIM should be close to 1, reflecting excellent structural and edge quality. Conversely, LABF and NABF should be close to zero, indicating minimal loss of edge information and artifacts introduced during the fusion process.

**Table 1:** Evaluation metrics of various methods on dataset-1.

Method	EN	PSNR	MI	AG	QABF	SSIM	LABF	NABF
LRD [6]	5.60	**35.22**	3.04	9.23	0.86	0.55	0.13	0.02
DTCWT [8]	5.41	34.26	2.40	9.74	0.87	0.21	0.11	0.02
CNN^†^ [13]	5.45	33.33	2.69	10.39	0.92	0.43	0.07	0.01
IMA [14]	**7.02**	17.44	3.39	9.92	0.92	0.41	0.07	0.01
EMD [7]	4.95	34.21	3.61	**10.79**	0.92	0.63	0.07	0.03
STAW [12]	4.83	30.52	**4.46**	10.64	0.93	0.72	0.06	0.01
NSCT [5]	5.48	32.61	3.28	10.71	0.92	0.62	0.06	0.01
FCDFusion^†^ [25]	4.45	33.47	4.31	6.3	0.69	0.68	0.29	0.02
CDDFuse^†^ [26]	5.35	33.82	3.48	10.66	**0.94**	**0.73**	**0.05**	**0.01**
CMTFusion^†^ [27]	6.59	34.83	2.87	6.31	0.65	0.28	0.34	0.01
Avg. (Higher is better)	5.51	31.97	3.35	9.47	0.86	0.53		
Avg. (Lower is better)							0.13	0.02
Proposed	4.72	34.64	3.19	10.12	0.91	0.66	0.08	0.02

The best values for each metric are highlighted in **bold**, and the second-best values are underlined. Higher values of EN, PSNR, MI, and AG indicate better performance. QABF and SSIM should be close to 1, while LABF and NABF should be close to 0.

† denotes deep learning-based methods.

The results in Table 1 demonstrate that the proposed technique achieves superior performance compared to learning-based methods such as CNN, FCDFusion, and CCDFuse in terms of PSNR, which reflects its ability to preserve image quality with minimal distortion. While CMTFusion achieves a marginally higher PSNR, the proposed technique outperforms it in all other critical metrics except EN. This indicates that the proposed method not only maintains high-quality image fidelity but also excels in preserving structural details (QABF, LABF, NABF, and SSIM) and enhancing image sharpness (AG). To better demonstrate its effectiveness, we have separately averaged the “higher-value-better” and “lower-value-better” quality indices across all methods and included these values in Table 1. The results show that the proposed method exceeds the average performance in 6 out of 8 quality metrics, underscoring its robustness for MRI-PET image fusion.

**Fig 9:**
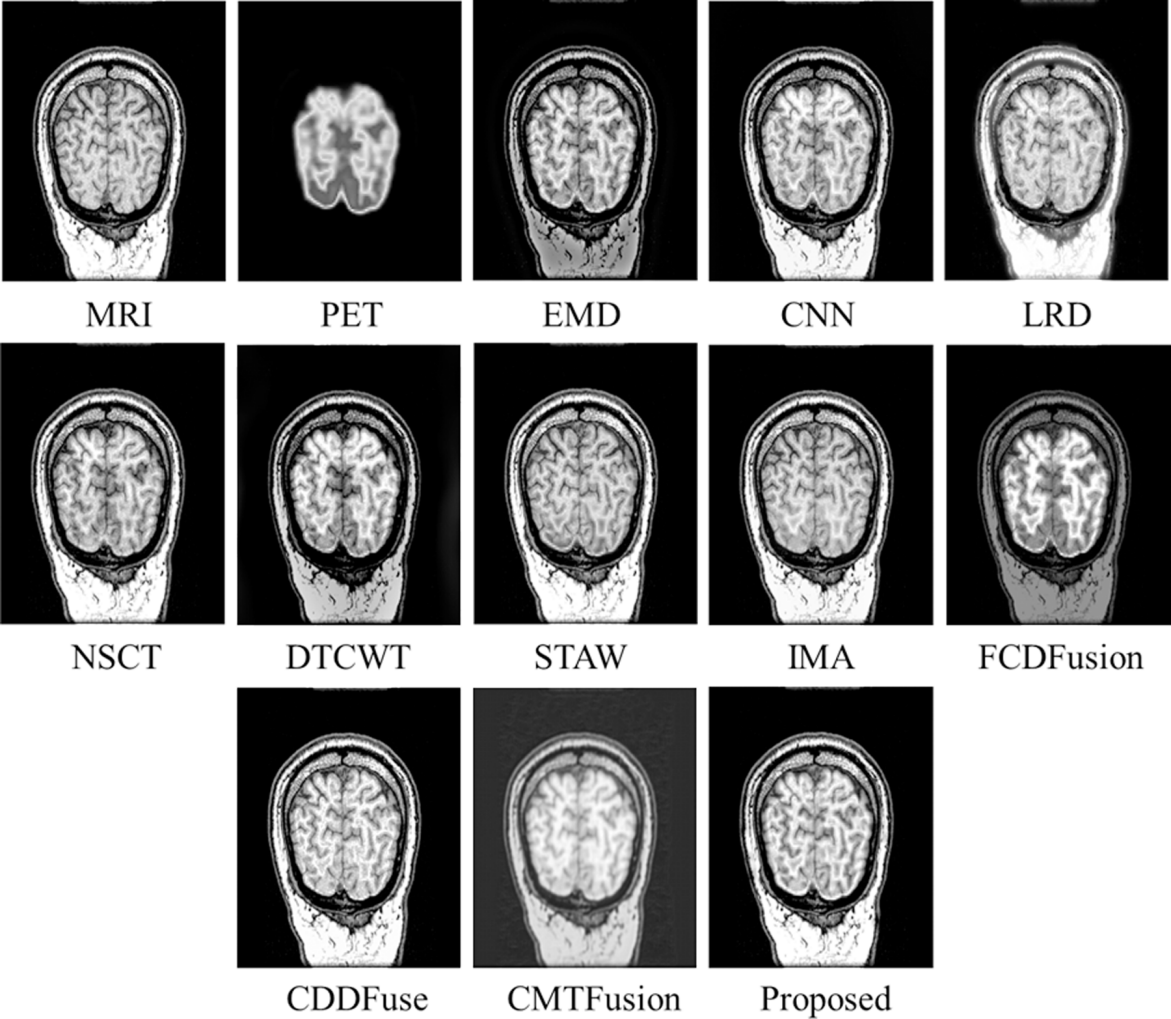
Fusion results of various techniques on a sample image pair from [29].

**Fig 10:**
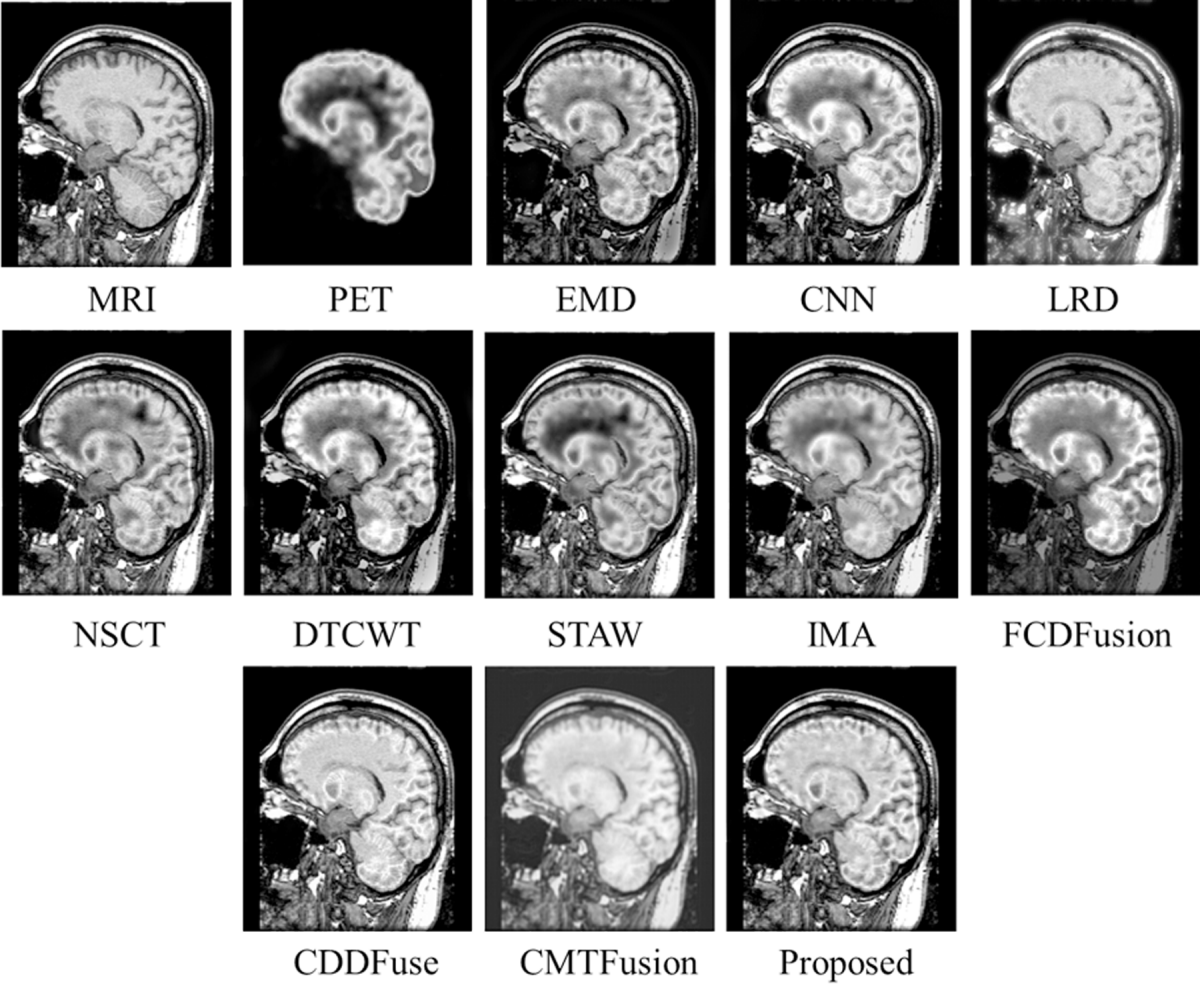
Fusion results of various techniques on a sample image pair from [29].

The fusion results of various methods on two sample image pairs taken from [29] are displayed in Figs 9 and 10 respectively. Table 2 presents the comparison of evaluation results. The proposed technique performs consistently well across multiple metrics, demonstrating its effectiveness in MRI-PET fusion. It achieves a PSNR of 32.298, which is comparable to methods like NSCT and EMD. Although CMTFusion achieves the highest PSNR, the proposed method significantly outperforms CMTFusion in almost all other parameters, such as QABF, SSIM, and AG, making it a better overall choice.

**Table 2:** Evaluation metrics of various methods on dataset-2.

Method	EN	PSNR	MI	AG	QABF	SSIM	LABF	NABF
LRD [6]	6.12	33.381	2.939	9.243	0.822	0.645	0.169	0.009
DTCWT [8]	5.509	33.274	2.295	10.324	0.877	0.615	0.103	0.02
CNN^†^ [13]	5.706	33.004	2.577	10.809	0.912	0.657	0.074	0.013
IMA [14]	5.465	29.662	3.727	10.710	0.907	0.702	0.092	**0.001**
EMD [7]	5.656	32.809	2.318	10.347	0.893	0.617	0.091	0.015
STAW [12]	5.145	31.889	4.218	11.170	**0.923**	0.704	0.071	0.005
NSCT [5]	5.599	32.286	3.162	11.293	0.915	0.702	0.071	0.014
FCDFusion^†^ [25]	5.185	32.714	**4.768**	7.399	0.724	0.668	0.259	0.017
CDDFuse^†^ [26]	5.615	31.604	3.68	**11.562**	0.919	**0.717**	**0.063**	0.018
CMTFusion^†^ [27]	**6.649**	**34.936**	2.527	6.763	0.656	0.27	0.34	0.004
Avg. (Higher is better)	5.665	32.556	3.221	9.962	0.855	0.63		
Avg. (Lower is better)							0.133	0.012
Proposed	5.387	32.298	3.269	10.878	0.915	0.714	0.073	0.012

*The best values for each metric are highlighted in* ***bold****, and the second-best values are underlined. Higher values of EN, PSNR, MI, and AG indicate better performance. QABF and SSIM should be close to 1, while LABF and NABF should be close to 0.*

† *denotes deep learning-based methods.*

Compared to learning-based methods like CNN, FCDFusion, and CDDFuse, the proposed technique shows superior performance in key metrics. It has a higher MI than CNN and better SSIM than FCDFusion and CNN, indicating its ability to retain critical information and structural details. While CDDFuse slightly outperforms the proposed technique in SSIM and AG, the proposed method provides a balanced trade-off across all metrics, including strong structural preservation and competitive sharpness. The proposed method outperforms the dataset-2 average in 6 out of 8 quality metrics, further highlighting its robustness and effectiveness.

When comparing the results from Table 1 and Table 2, the proposed technique consistently delivers balanced and robust performance across diverse datasets. While other methods may excel in specific metrics or datasets, the proposed technique ensures reliable fusion quality by maintaining a strong overall performance. This consistency across both datasets reinforces its suitability for MRI-PET fusion in practical medical applications.

Table 3 presents the run times (in seconds) of various methods averaged over 34 images, providing insights into the computational efficiency of each technique. The proposed method achieves an exceptionally low run time of 0.020 seconds, making it significantly faster than most other methods listed. Overall, the proposed technique not only delivers high-quality fusion results but also ensures fast processing times, making it an ideal choice for real-time applications where both performance and speed are critical.

**Table 3:** Average run times (in seconds) for different methods.

Method	Time	Method	Time	Method	Time
LRD [6]	24.57	EMD [7]	19.94	CDDFuse^†^ [26]	0.21
DTCWT [8]	0.025	STAW [12]	0.03	CMTFusion^†^ [27]	0.08
CNN^†^ [13]	7.34	NSCT [5]	1.27	Proposed	**0.020**
IMA [14]	0.11	FCDFusion^†^ [25]	0.75		

† denotes deep learning-based methods.

To assess the effectiveness of the proposed method, we conducted an ablation study, summarized in Table 4. The study was divided into four phases to evaluate the contribution of each component. In the first phase, we used only the LP and mean fusion as a baseline, focusing on the multi-resolution decomposition of images. The second phase incorporated EMD with mean fusion, enabling finer decomposition of high-frequency components into IMFs and residues. The third phase replaced mean fusion with PC for fusing IMFs, emphasizing subtle intensity variations and structural details in high-frequency components. Finally, the fourth phase combined all components—EMD, PC, and the RGF. The RGF significantly enhanced edge preservation and minimized artifacts, resulting in the most robust fusion performance. This phase represents the full implementation of the proposed method.

**Table 4:** Ablation study of the proposed fusion framework.

	EN	PSNR	MI	QABF	LABF	NABF	AG	SSIM
**Phase 1**	5.045	29.311	2.132	0.822	0.168	0.019	9.253	0.645
**Phase 2**	5.101	31.909	2.319	0.893	0.092	0.015	10.223	0.681
**Phase 3**	5.356	32.108	2.318	0.915	0.081	0.015	10.247	0.709
**Phase 4**	**5.387**	**32.298**	**3.269**	**0.915**	**0.073**	**0.012**	**10.878**	**0.714**

## 5. Conclusion

In conclusion, the proposed MRI-PET fusion technique utilizing LP and EMD marks a significant advancement in oncology imaging. By effectively merging the high-resolution anatomical data from MRI with the functional insights from PET scans, this method enhances the diagnostic capabilities far beyond what is possible with traditional fusion techniques. Our comprehensive evaluation demonstrates that the method not only preserves essential structural and edge details but also significantly improves objective metrics such as PSNR, MI, and SSIM compared to existing methods. This approach sets a new standard for high-quality, efficient medical image processing, potentially transforming diagnostic procedures in oncology by providing clearer, more informative images that facilitate better patient outcomes.
